# A Bibliometric and Knowledge-Map Analysis of Macrophage Polarization in Atherosclerosis From 2001 to 2021

**DOI:** 10.3389/fimmu.2022.910444

**Published:** 2022-06-20

**Authors:** Luxia Song, Jie Zhang, Dan Ma, Yixuan Fan, Runmin Lai, Wende Tian, Zihao Zhang, Jianqing Ju, Hao Xu

**Affiliations:** ^1^ Graduate School, Beijing University of Chinese Medicine, Beijing, China; ^2^ National Clinical Research Center for Chinese Medicine Cardiology, Xiyuan Hospital, China Academy of Chinese Medical Sciences, Beijing, China; ^3^ Graduate School, China Academy of Chinese Medical Sciences, Beijing, China

**Keywords:** macrophage polarization, atherosclerosis, knowledge-map, CiteSpace, VOSviewer

## Abstract

In recent years, studies of macrophage polarization in atherosclerosis have become an intense area of research. However, there are few bibliometric analyses regarding this area. In this review, we used CiteSpace 5.8.R3 and VOSviewer 1.6.16 software to perform text mining and knowledge-map analysis. We explored the development process, knowledge structure, research hotspots, and potential trends using a bibliometric and knowledge-map analysis to provide researchers with a macroscopic view of this field. The studies concerning macrophage polarization in atherosclerosis were downloaded from the Web of Science Core Collection. A total of 781 studies were identified and published by 954 institutions from 51 countries/regions. The number of studies of macrophage polarization in atherosclerosis increased over time. *Arteriosclerosis Thrombosis and Vascular Biology* published the highest number of articles and was the top co-cited journal. De Winther was the most prolific researcher, and Moore had the most co-citations. The author co-occurrence map illustrated that there was active cooperation among researchers. The most productive countries were the United States and China. Amsterdam University, Harvard University, and Maastricht University were the top three productive institutions in the research field. Keyword Co-occurrence, Clusters, and Burst analysis showed that “inflammation,” “monocyte,” “NF kappa B,” “mechanism,” and “foam cell” appeared with the highest frequency in studies. “Oxidative stress,” “coronary heart disease,” and “prevention” were the strongest citation burst keywords from 2019 to 2021.

## Introduction

Atherosclerosis (AS) is a complex chronic inflammatory disease that occurs in the arterial vessel wall, involving large- and medium-sized muscular arteries, and contributes to cardiovascular and cerebrovascular mortality. Plaque growth leads to arterial lumen narrowing and plaques rupture, causing ischemia, necrosis, and hemorrhage in the tissues or organs supplied by the artery. In atherosclerotic lesions, there is intimal thickening, lipid deposition, and infiltration of monocytes and lymphocytes. Smooth muscle cells migrate and proliferate in the intima, secreting extracellular matrix components such as collagen. Macrophages differentiated from monocytes take up oxidized low-density lipoprotein (ox-LDL) to form foam cells (1). Foam cells are aggravated and lead to the formation of a lipid necrotic core, which was covered with a fibrous cap.

Macrophages are the primary inflammatory cells in plaques that promote early plaque formation, fibrous cap dilution, and necrotic core formation and enhance immune response (2). Macrophages undergo phenotypic transformation (i.e., macrophage polarization) under the influence of several factors. M1-type macrophages (also known as classically activated macrophages) can be generated by stimulating inflammatory factors such as lipopolysaccharide, gamma interferon, and granulocyte macrophage colony-stimulating factor. Pro-inflammatory factors tumor necrosis factor-α (TNF-α), interleukin (IL)–6, IL-1β, C-C Motif Chemokine Ligand 5 (CCL-5), and inducible nitric oxide synthase are secreted by M1 cells and participate in inflammatory response initiation and maintenance (3, 4). M2-type macrophages (also known as alternatively activated macrophages) are produced by stimulation with the macrophage colony-stimulating factor, IL-4, and IL-13. They secrete the anti-inflammatory cytokines IL-10, IL-1 receptor antagonist, and CCL18. These macrophages express arginase 1 and Fizz1 (5). M2 macrophages are involved in tissue remodeling (6) and inflammation regression (7). The ratio of M1/M2 determines the development trend and stability of atherosclerotic plaques. Increased M1 macrophages leads to the secretion of inflammatory factors and causes endothelial cell dysfunction, resulting in fibrous cap dilution. Increased M2-type macrophages phagocytose apoptotic M1 cells, prevent plaque rupture, and inhibit AS (8). The concept of macrophage polarization has provided a basis for the study of inflammatory immunity in atherosclerotic plaques, and there are several lines of evidence, suggesting that macrophage polarization participates in plaque formation and its stability. However, macrophage subtypes are not limited to M1 and M2. The origin and characteristics of some macrophages that are closely associated with human disease development remain unclear, including CD169+ macrophages, TCR+ macrophages, and tumor-associated macrophages (9).

We applied the most commonly used bibliometric software packages (CiteSpace and VOSviewer) to analyze and visualize macrophage polarization’s knowledge base and potential trends in AS research. First, we identified the annual outputs, author impacts, cooperation, countries/regions, institutions, and journal-related information to determine the general information in this field. Second, we evaluated the knowledge base on research topics using an analysis of co-cited references. Third, keyword detection (including co-occurrence and cluster analysis) was used to detect hotspots and their evolution from 2001 to 2021. Whereas keywords and co-cited reference burst analysis were used to identify emerging topics.

## Methods

### Data Acquisition

The scientific studies were downloaded from the Web of Science Core Collection (WoSCC) database on February 15, 2022. Search session Queries: TS = (macrophage polarization) AND ((((((((((((((TS = (Arteriosclerosis)) OR TS = (Atheromatous Plaques)) OR TS = (Atheromatous Plaque)) OR TS = (Fibroatheroma)) OR TS = (Fibroatheromas)) OR TS = (Arterial Fatty Streak)) OR TS = (Arterial Fatty Streaks)) OR TS = (Atherosclerotic Plaques)) OR TS = (Atherosclerotic Plaque)) OR TS = (Atheroma)) OR TS = (Atheromas)) OR TS = (Atheromatous Plaques)) OR TS = (Atheromatous Plaque)) OR TS = (Atherosclerosis)) OR TS = (Arteriosclerosis); Publication date: “2010-01-01” to “2021-12-31”; Document types: articles an review articles. The search results were exported with “Plain Text file” and the record content chose “Full Record and Cited References,” and stored in download_*.txt format.

### Data Analysis and Visualization

CiteSpace is a Java application for bibliometric analysis developed by Chen (10). It enables knowledge mining and visualization in bibliographic databases, aiming to explore author, countries and institutional cooperation, knowledge domain, the emergence of subjects, and the future research trends (11). We used CiteSpace 5.8.R3 to detect and visualize the author, countries/regions and institutions collaboration, keywords co-occurrence, cluster and burst, co-cited references, and citation burst. We imported the “download_*.txt” file into CiteSpace 5.8.R3 and selected “Data” to remove duplicated studies. The time span was set as 2001–2021.12 and years per slice; Top N = 50 filtered the top 50 authors, organizations, and keywords with the highest frequency in each time slice. The network pruning was based on the preliminary analysis results to choose Pathfinder Network (PFNET), Minimum Spanning Tree (MST), or no network pruning. In the keyword co-occurrence analysis, we merged the synonyms to an alias list, including “M1 macrophage” and “M1,” and removed nonsense words like “alpha” and “pet.” In the institutions and author analysis, the same institutions and authors with different spellings (e.g., “Washington Univ” and “Univ Washington,” “Stephen Sansom,” and “Stephen N Sansom”) were also merged.

In 2009, Eck and Waltman from Leiden University constructed a program used for developing a scientometrics network and knowledge-map visualization called VOSviewer (12). VOSviewer has an advantage in handling large bibliometric maps and builds co-citation maps for major journals. We used VOSviewer to identify productive journals and co-cited journals. Import studies were retrieved from WoSCC and analyzed with VOSviewer 1.6.16 based on the full counting method, which means each co-citation link or co-occurrence could have the same weight. In productive journal analysis, the minimum number of documents per journal was set at 5; and in co-cited journals analysis, the minimum number of citations per source was set at 20. Annual outputs were managed using Microsoft Office Excel 2019 to show research trends in this area. In addition, the 2020 journal impact factor (IF) and JCR were obtained from the Web of Science.

## Results

### Annual Trend of Publications

**Figure 1:**
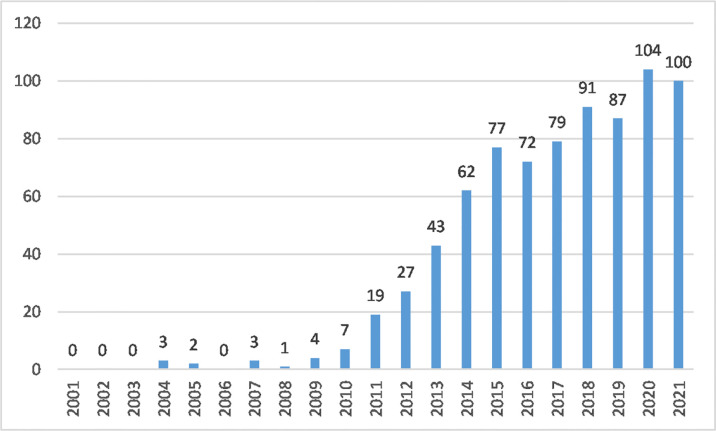
Annual output of macrophage polarization in AS research.

From 2001 to 2021, 781 articles and review articles were published in this field. The annual growth of outputs demonstrated the trends of research. **Figure 1** shows that the number of published studies was low from 2001 to 2010.

From 2011 to 2015, as more scholars focused on AS mechanisms, the annual outputs of macrophage polarization in this area increased rapidly. Articles published in 2015 reached 77. By the end of 2021, research articles reached 104 (2020) and 100 (2021), showing steady growth.

### Authors and Co-Cited Authors

In 1997, the metrologists Katz and Martin (13) defined “scientific collaboration” as scholars working together for common scientific objectives. CiteSpace identified 5,144 authors with published studies on macrophage polarization in AS. De Winther published the highest number of studies (n = 17), followed by Staels (n = 10), Fisher (n = 10), Chinetti-Gbaguidi (n = 9), and Daemen (n = 8) (**Table 1**).

**Table 1:** Top 10 authors and co-cited authors of macrophage polarization in AS research.

Rank	Author	Count	Co-cited author	Co-citation
1	Menno P.J. de Winther	17	Mantovani A	221
2	Bart Staels	10	Moore KJ	217
3	Edward A Fisher	10	Gordon S	196
4	Giulia Chinettigbaguidi	9	Martinez FO	186
5	Mat J.A.P. Daemen	8	Libby P	180
6	Marion J Gijbels	8	Tabas I	159
7	Reto Asmis	7	Murray PJ	154
8	Esther Lutgens	7	Chinettigbaguidi G	142
9	Sina Tavakoli	7	Mosser DM	136
10	Erik A L Biessen	7	Stoger JL	132

**Figure 2:**
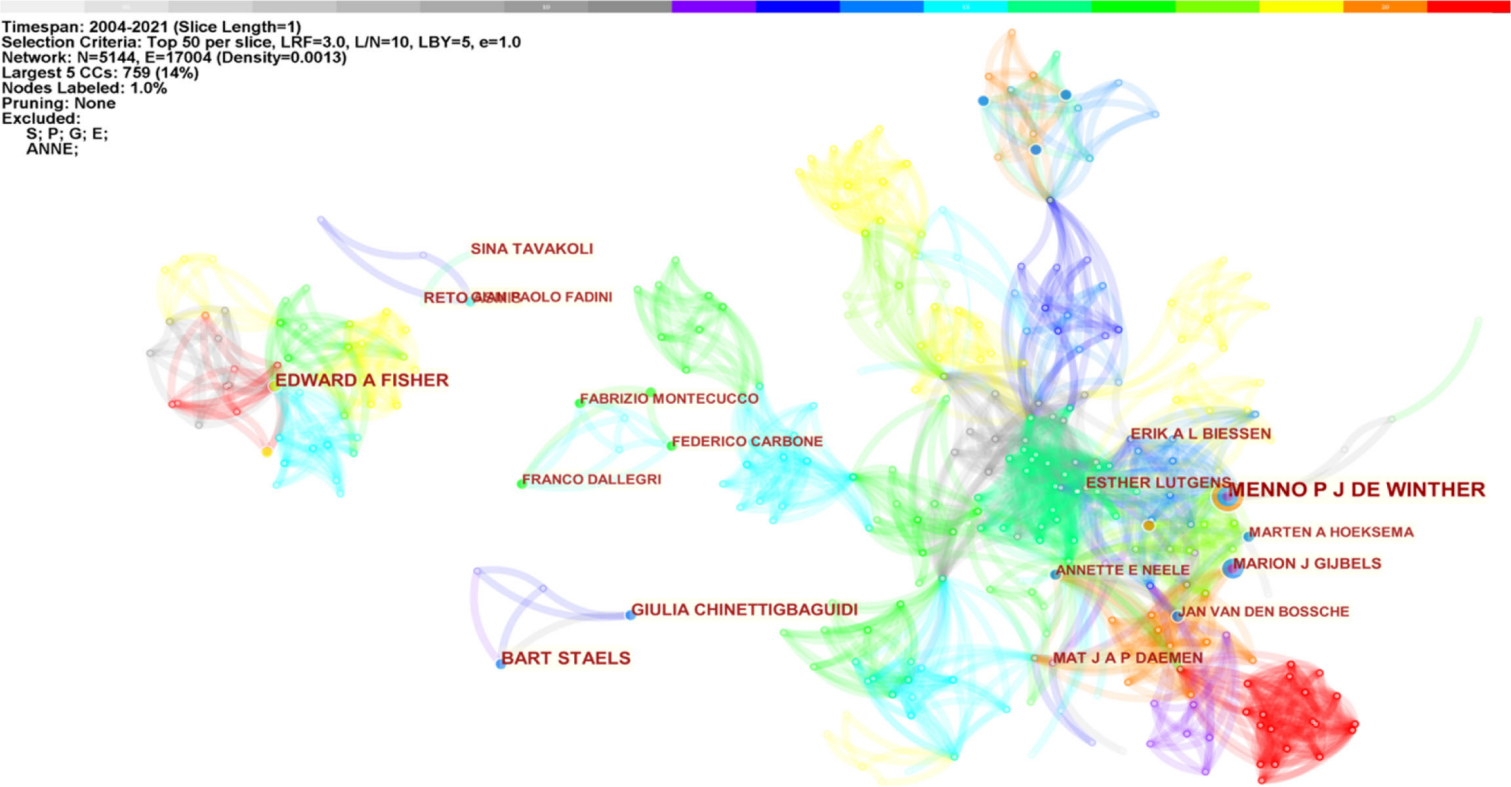
Author co-occurrence map of macrophage polarization in AS research (K = 5).

Betweenness centrality measures critical nodes in bibliometric maps (10). Authors in the co-occurrence map showed low centrality (= 0), indicating that scholars need to engage in further exploration and collaboration on this topic. **Figure 2** shows the five most significant connected components (k = 5) of author co-occurrence, containing 5,144 network nodes and 17,004 connections. Network nodes represent authors, and the size of the nodes is proportional to the number of studies posted by them. Link colors vary with the years articles were published, and link clusters represent author cooperation relationships. De Winther, Lutgens, Biessen, Daemen, Gijbels, Neele, and Van den Bossche closely collaborated in macrophage polarization research in AS.

**Figure 3:**
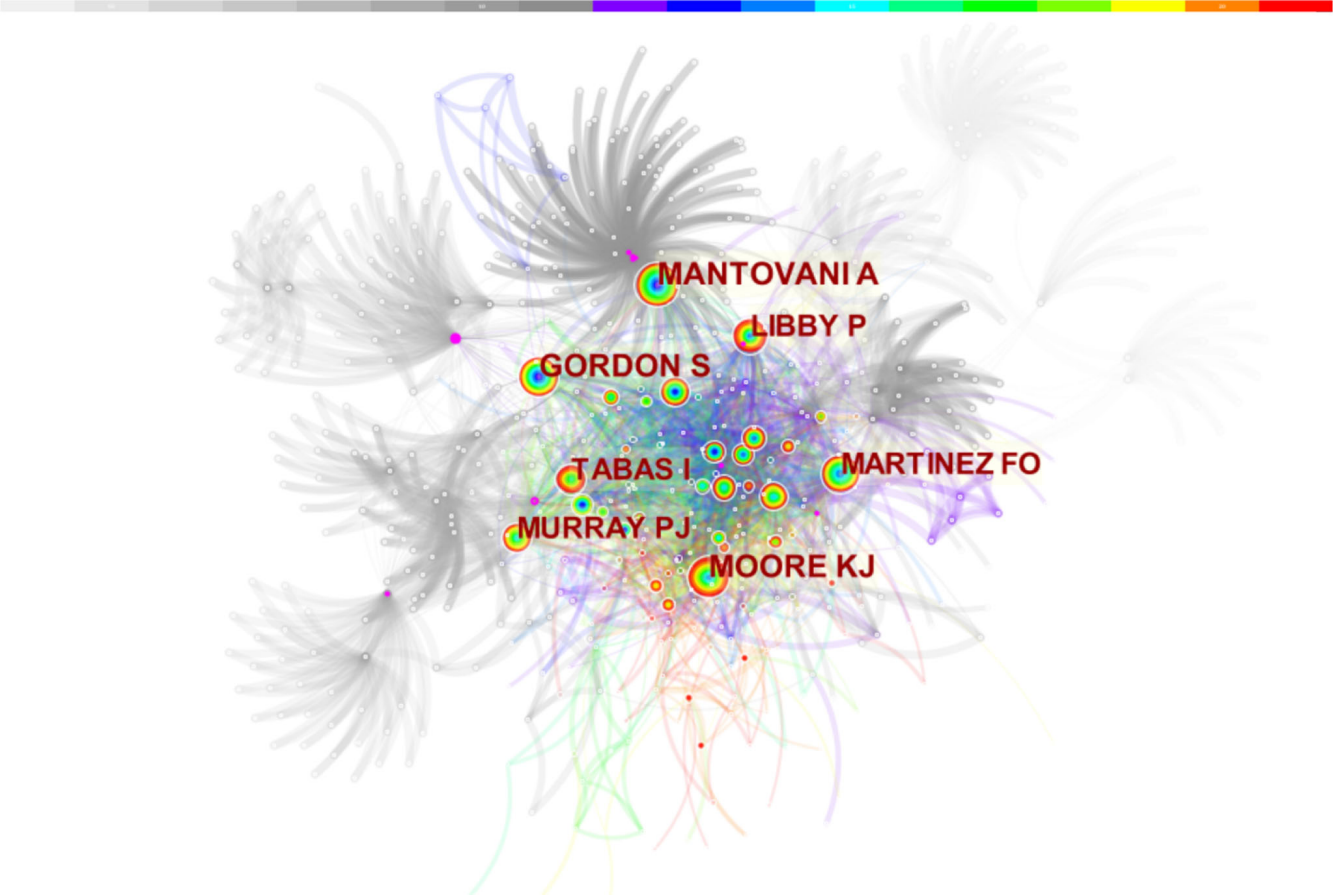
Co-cited author co-occurrence map of macrophage polarization in AS research.

Co-cited authors refer to authors cited simultaneously in articles (**Figure 3**). Of the 853 co-cited authors, 27 were co-cited over 50 times, and only two authors were cited over 200 times. The top five co-cited authors were Mantovani (n = 221), Moore (n = 217), Gordon (n = 196), Martinez (n = 186), and Libby (n = 180) (**Table 1**).

### Countries/Regions and Institutions Co-Occurrence

**Figure 4:**
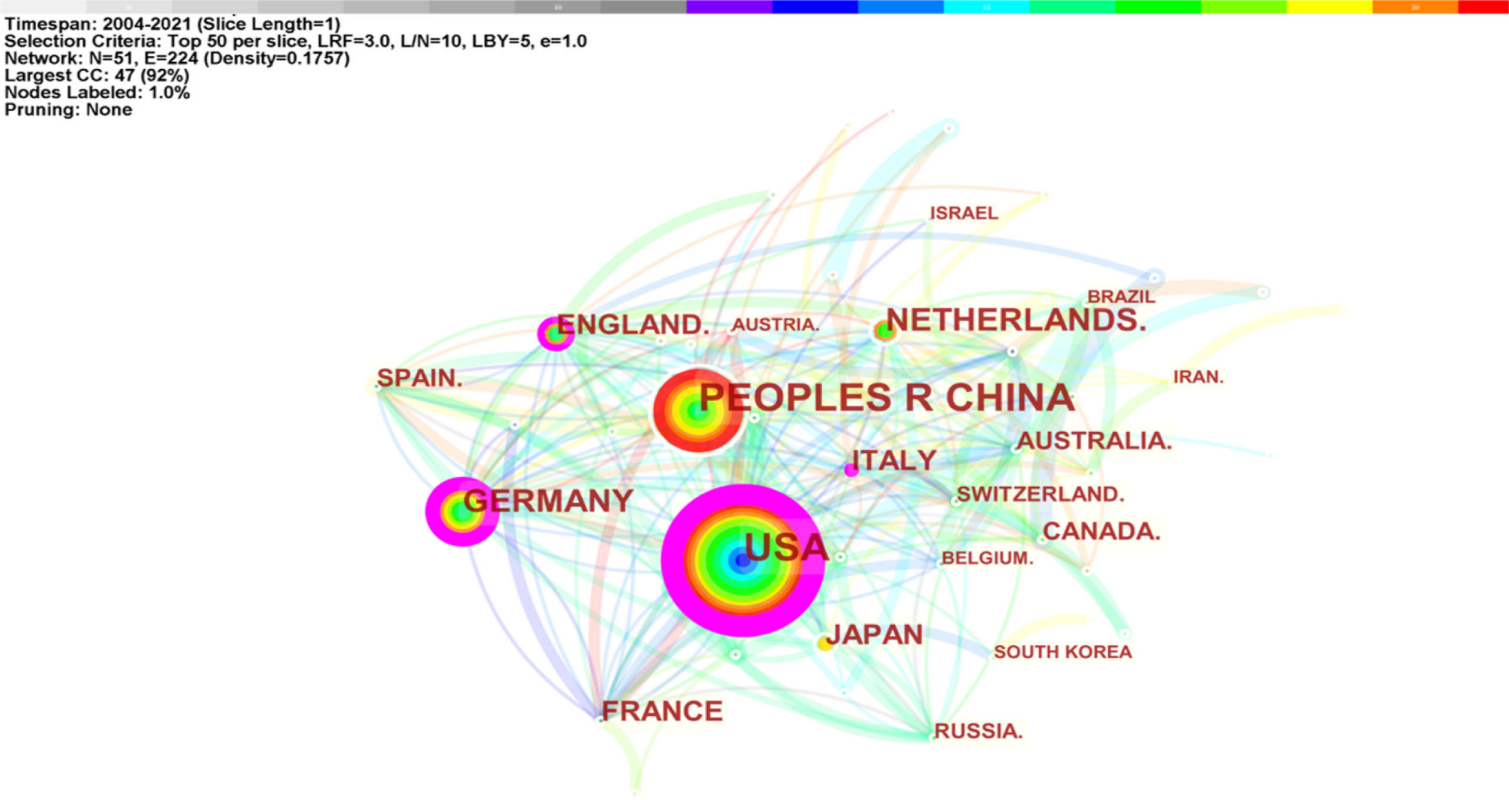
Country co-occurrence map of macrophage polarization in AS research (T ≥ 20).

There were 781 studies published by 954 institutions from 51 countries/regions. The United States (242, 30.98%) published the most significant number of articles on macrophage polarization in AS, followed by China (227, 29.07%), Germany (154, 8.60%), and Japan (103, 5.75%) (**Table 2**). As shown in **Figure 4**, the largest connected components of countries/regions co-occurrence contained 51 nodes and 224 connections with a map density of 0.1757. The nodes colored with purple showed the betweenness centrality of countries/regions higher than 0.10, including the United States (0.53), Germany (0.27), Italy (0.15), and England (0.14), meaning that these countries/regions took “bridge” roles in this field.

**Table 2:** The top 10 countries/regions and institutions of macrophage polarization in AS.

Rank	Country/region	Year	N (%)	Centrality	Institution	Country/region	N (%)	Centrality
1	USA	2004	242 (30.99%)	0.53	Amsterdam University	Netherlands	21 (2.20%)	0.05
2	China	2013	227 (29.07%)	0.08	Harvard University	USA	21 (2.20%)	0.14
3	Germany	2010	75 (9.60%)	0.27	Maastricht University	Netherlands	21 (2.20%)	0.11
4	Netherlands	2010	55 (7.04%)	0.08	IRCCS	Italy	19 (1.99%)	0.06
5	Japan	2007	53 (6.79%)	0.03	Huazhong University of Science and Technology	China	18 (1.89%)	0.05
6	Italy	2005	51 (6.53%)	0.15	New York University	USA	18 (1.89%)	0.17
7	England.	2010	42 (5.38%)	0.14	INSERM	France	15 (1.57%)	0.09
8	France	2009	37 (4.74%)	0.08	Shanghai Jiao Tong University	China	14 (1.47%)	0.06
9	Australia	2012	24 (3.07%)	0.06	Harbin Medical University	China	14 (1.47%)	0.02
10	Spain	2011	21 (2.69%)	0.1	Washington University	USA	14 (1.47%)	0.06
11	Canada.	2009	21 (2.69%)	0.02	UCL	England	14 (1.47%)	0.22

**Figure 5:**
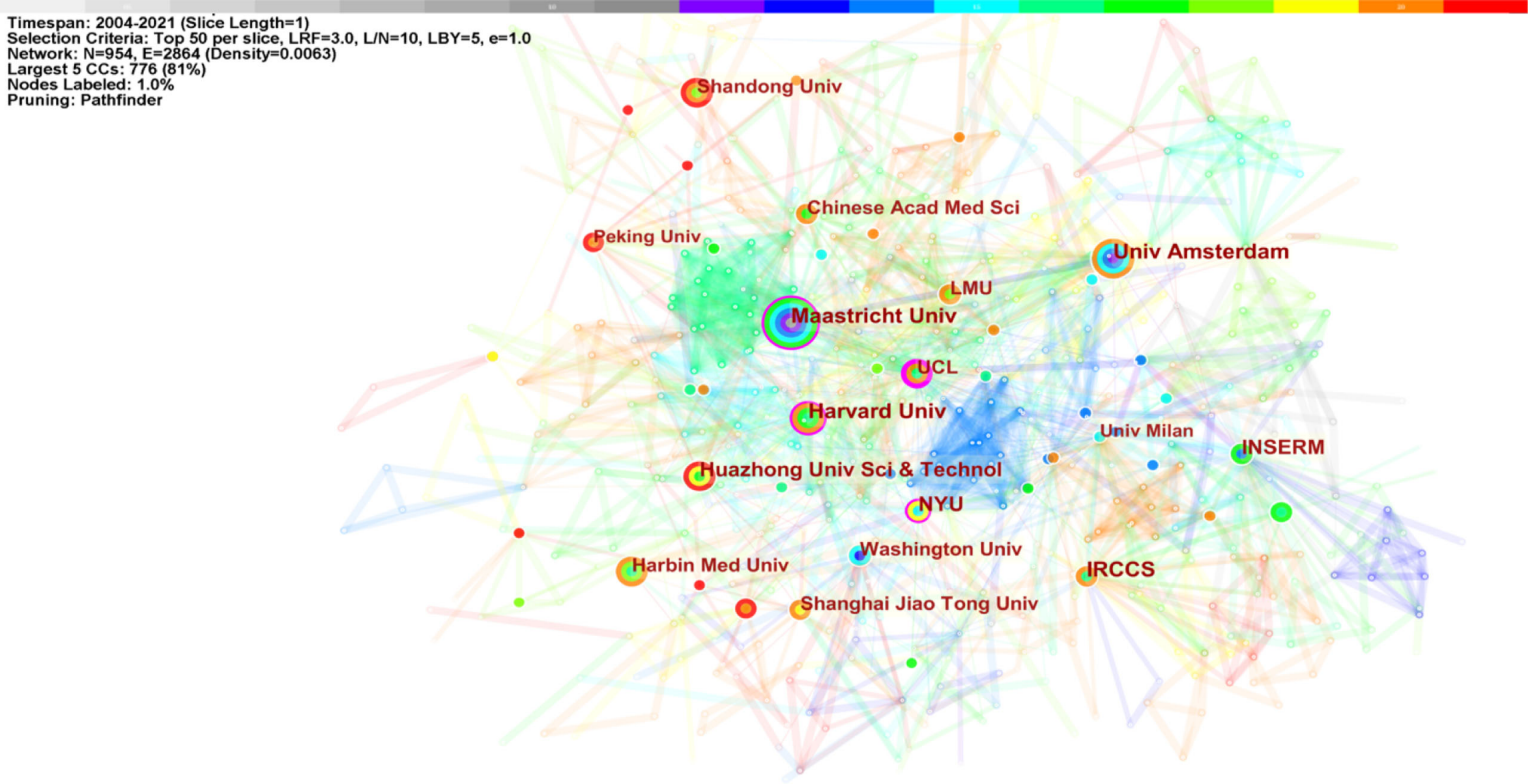
Institution co-occurrence map of macrophage polarization in AS research (K = 5).

The top 11 institutions were from China (3/10), the USA (3/10), the Netherlands (2/10), Italy (1/10), and France (1/10). Amsterdam University (21, 2.20%), Harvard University (21, 2.20%), Maastricht University (21, 2.20%), IRCCS (19, 1.99%), and Huazhong University of Science and Technology (18, 1.89%) were the top five productive institutions (**Table 2**). **Figure 5** displays the five largest connected components (k = 5) with pathfinder-pruning institutions co-occurrence, containing 954 network nodes and 2,864 connections (Density = 0.0063); UCL (14, 1.47%) presented the highest centrality (0.22), followed by New York University (0.17), Harvard University (0.14), and Maastricht University (0.11), whose nodes were identified with purple circles. Countries/regions and institutions engaged in frequent collaborations (**Figures 4**, **5**).

### Journals and Co-Cited Journals

VOSviewer 1.6.16 was used to construct journal co-citation and co-cited analysis; 781 references concerning macrophage polarization research in AS were published in 317 academic journals, of which 35 journals had an IF over 5. *Arteriosclerosis Thrombosis and Vascular Biology* published the highest number of studies (41, 5.25%), followed by Atherosclerosis (30, 3.84%), *Frontiers in Immunology* (26, 3.33%), Plos One (26, 3.33%), and *Scientific Reports* (21, 2.69%). *Circulation Research* had the highest IF of 17.367. Among the top 10 journals, 6 (*Arteriosclerosis Thrombosis and Vascular Biology*, *Frontiers in Immunology*, *Scientific Reports*, *International Journal of Molecular Sciences*, *Circulation Research*, and *Frontiers in Pharmacology*) were at the Q1 JCR division, and six had an IF of more than 5 (**Table 3**).

**Table 3:** The top 10 journals of macrophage polarization in AS research.

Rank	Journal	N (%)	IF (2020)	JCR (2020)
1	Arteriosclerosis Thrombosis and Vascular Biology	41 (5.25%)	8.313	Q1
2	Atherosclerosis	30 (3.84%)	5.162	Q2/Q1
3	Frontiers in Immunology	26 (3.33%)	7.561	Q1
4	Plos One	26 (3.33%)	3.24	Q2
5	Scientific Reports	21 (2.69%)	4.38	Q1
6	International Journal of Molecular Sciences	16 (2.05%)	5.924	Q1/Q2
7	Biochemical and Biophysical Research Communications	14 (1.79%)	3.575	Q3/Q2
8	Circulation Research	12 (1.54%)	17.367	Q1
9	Current Opinion in Lipidology	11 (1.41%)	4.776	Q2
10	Frontiers in Pharmacology	11 (1.41%)	5.811	Q1

VOSviewer found 3,087 co-cited journals in the past 20 years. Nineteen journals had citations over 500, and eight journals had over 1000. As is shown in **Table 4**, *Arteriosclerosis Thrombosis and Vascular Biology* was the most co-cited journal (2,821), followed by *Circulation Research* (1,490), *Journal of Immunology* (1,381), Circulation (1,378), and Journal of Clinical Investigation (1,360). Nature had the highest IF (49.962) among the top 10 co-cited journals, followed by *Circulation* (29.69). Seven of nine co-cited journals were in the Q1 district of JCR, and the remainder were in Q2.

**Table 4:** The top 10 co-cited journals of macrophage polarization in AS research.

Rank	Co-cited Journal	N	IF (2020)	JCR (2020)
1	Arteriosclerosis Thrombosis and Vascular Biology	2128	8.313	Q1
2	Circulation Research	1490	17.367	Q1
3	Journal of Immunology	1381	5.422	Q2
4	Circulation	1378	29.69	Q1
5	Journal of Clinical Investigation	1360	14.808	Q1
6	Journal of Biological Chemistry	1346	5.157	Q2
7	Proceedings of the National Academy of Sciences of the United States of America	1027	11.205	Q1
8	Nature	1001	49.962	Q1
9	Atherosclerosis	982	5.162	Q2/Q1
10	Plos One	932	3.24	Q2

### Keyword Co-Occurrence, Clusters, and Burst

**Figure 6:**
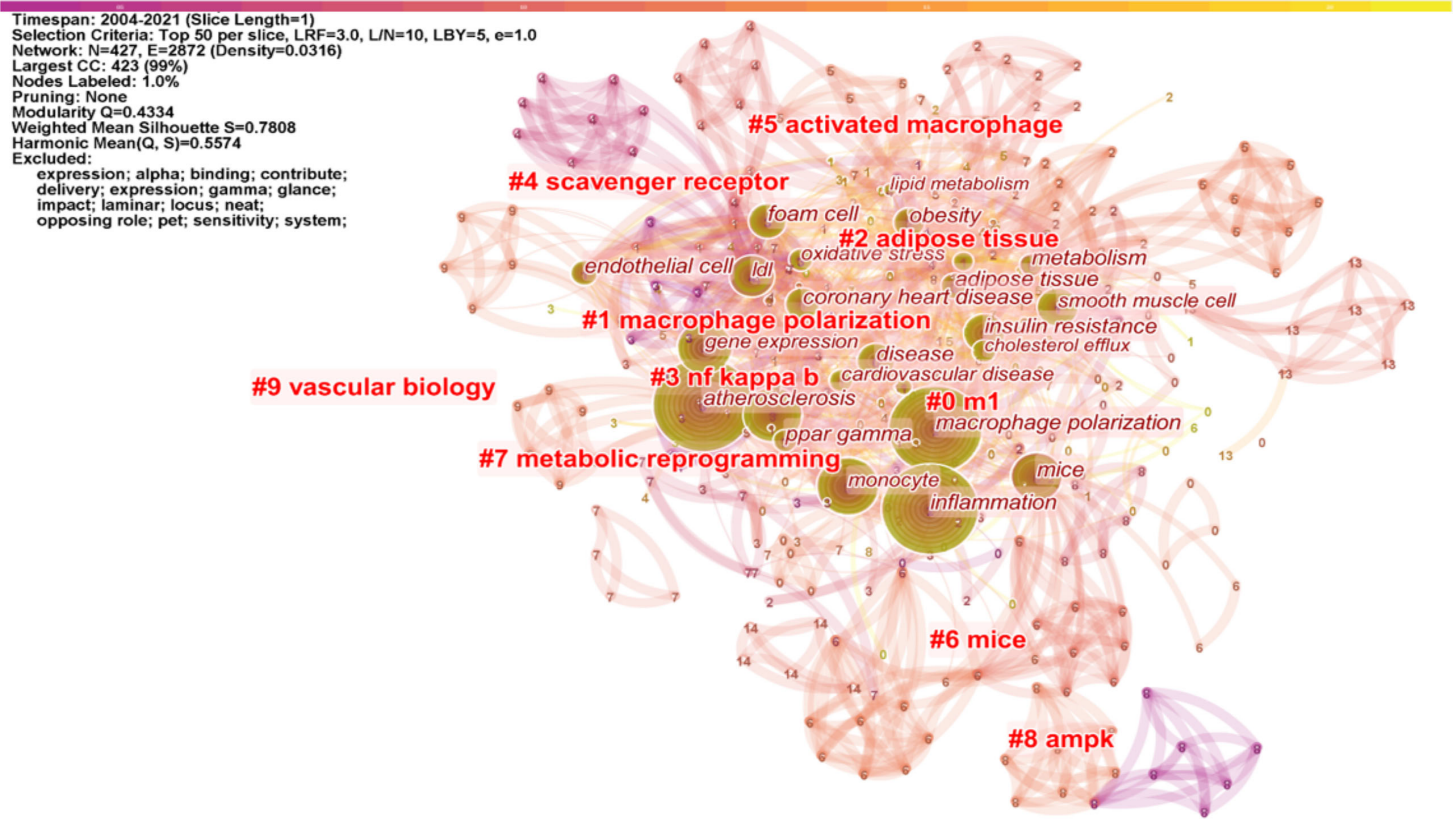
Keyword co-occurrence map and cluster of macrophage polarization in AS research.

CiteSpace was used to construct a keyword co-occurrence map (**Figure 6**). A total of 427 keywords were extracted, of which 72 terms appeared more than 10 times, and 18 appeared more than 50 times. **Table 5** shows the top 20 keyword co-occurrence terms. “Inflammation” (223), “monocyte” (94), “NF kappa b” (77), “mechanism” (223), and “foam cell” (50) were core contents of macrophage polarization research in AS. “Cell” (0.15), “mice” (0.12), “mechanism” (0.1), and “LDL” (0.1) shared “bridge” effects in the keyword co-occurrence map.

**Table 5:** Top 20 keywords of macrophage polarization in AS research.

Rank	Keyword	Count	Centrality	Rank	Keyword	Count	Centrality
1	atherosclerosis	273	0.05	11	alternative activation	67	0.01
2	macrophage polarization	253	0.06	12	differentiation	61	0.04
3	inflammation	223	0.05	13	insulin resistance	59	0.08
4	activation	184	0.07	14	mechanism	55	0.1
5	cell	111	0.15	15	disease	53	0.03
6	monocyte	94	0.03	16	smooth muscle cell	52	0.03
7	gene expression	82	0.04	17	LDL	50	0.1
8	mice	78	0.12	18	foam cell	50	0.07
9	NF kappa B	77	0.01	19	phenotype	49	0.03
10	macrophage	68	0.07	20	receptor	47	0.06

Cluster analysis is a statistical method of classifying data according to the degree of similarity, aiming to discover the distribution of research content on particular subjects (14). Modularity (Q-score) and Silhouette (S-score) evaluate the cluster mapping. Q > 0.3 means the structure of the delineated associations is significant; S > 0.5 means that the cluster is reasonable, and S > 0.7 indicates the cluster is efficient and convincing. We clustered the keywords using CiteSpace software. **Figure 6** (Q = 0.4334, S = 0.7808) displays the largest connected components with no-pruning keyword co-occurrence and cluster map, containing 427 nodes and 2,864 connections (Density = 0.0316). Fifteen clusters were extracted and labeled by “#” in the cluster map (**Figure 6** shows the top 10 clusters). Cluster #0 labeled theme by “m1” contained 85 co-occurrence keywords: activation, cell, differentiation, mechanism, disease, phenotype, proliferation, immune response, apoptosis, and m1 macrophage. The #1 macrophage polarization cluster included 53 keywords: smooth muscle cell, endothelial cell, coronary heart disease, cholesterol efflux, monocyte subset, and macrophage activation. The #2 cluster was related to adipose tissue, which included 52 keywords: inflammation, insulin resistance, obesity, PPAR gamma, adipose tissue macrophage, and metabolic. Cluster #3 focused on the NF kappa b topic and had 43 keywords in the cluster, including atherosclerosis, monocyte, foam cell, dendritic cell, oxidative stress, and shear stress. The #4 scavenger receptor cluster contained 35 keywords, including gene expression, LDL, lipid metabolism, CD36, MAPK, and innate immunity.

**Figure 7:**
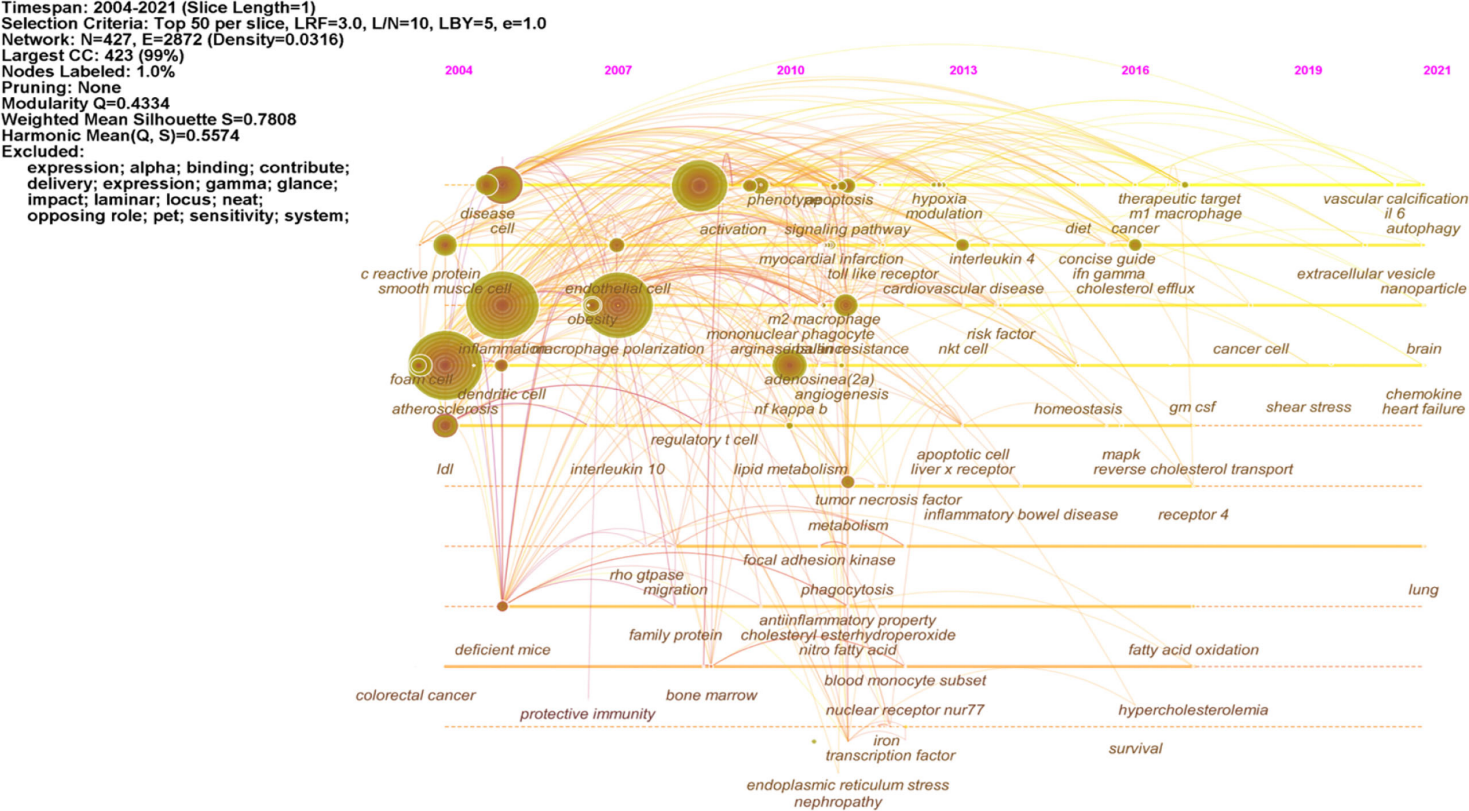
Keyword timeline view of macrophage polarization in AS research.

The keywords timeline view displays the evolution of high-frequency keywords. **Figure 7** shows the research hotspot progression path evolution in macrophage polarization in AS. From 2004 to 2010, research keywords in this area focused on foam cell, inflammation, endothelial cell, obesity, lipid metabolism, phagocytosis, and myocardial infarction. From 2011 to 2021, the primary terms were cardiovascular disease, cholesterol efflux, therapeutic target, fatty acid oxidation, shear stress, autophagy, and nanoparticle.

**Figure 8:**
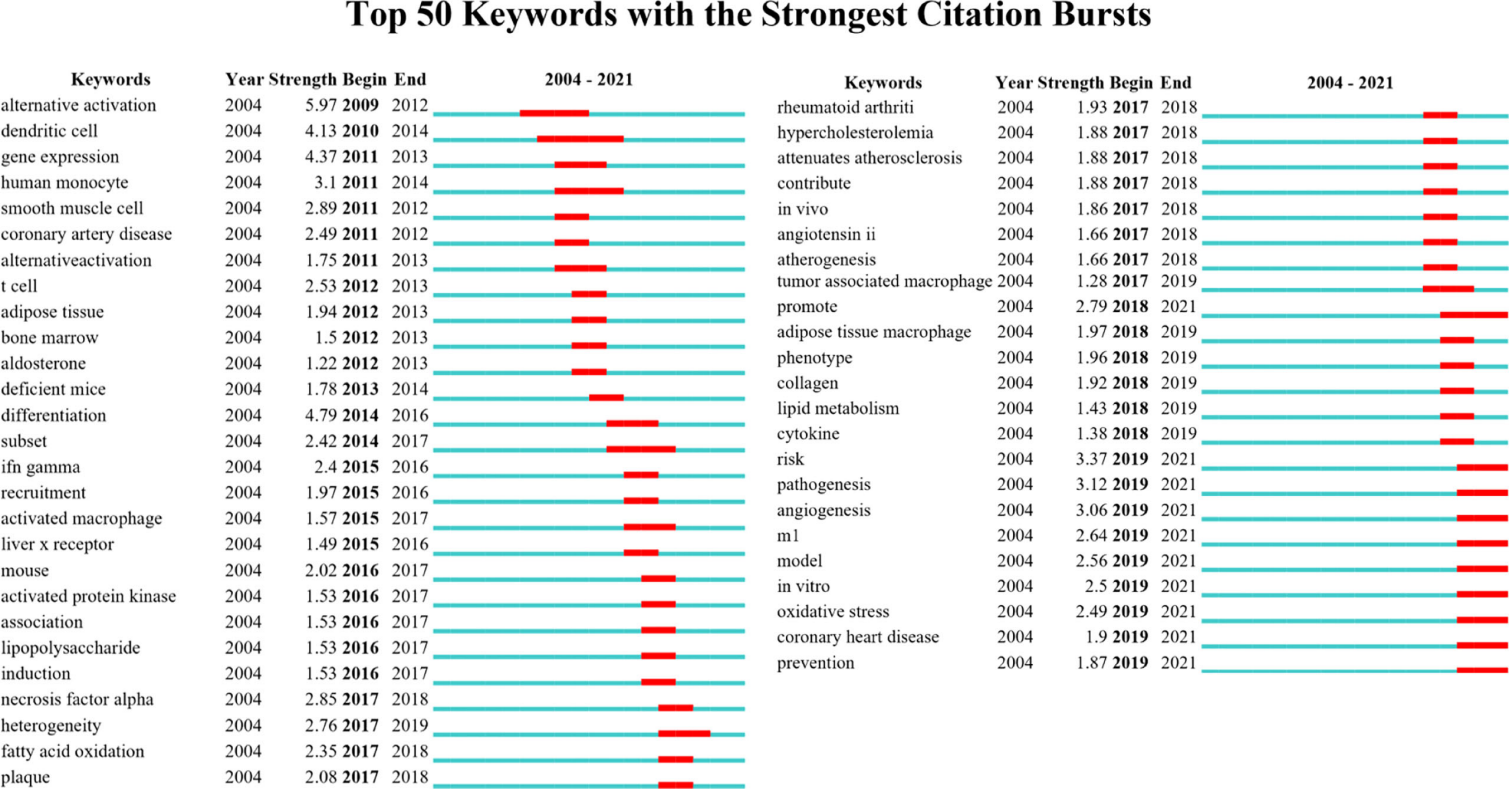
Top 50 keywords with citation burst (sorted by the beginning year of the burst).

Keywords burst detection identifies sudden increases in frequency within a short period, revealing research hotspots over time and reflecting the trend of hotspot evolution. The top 50 keywords with citation burst are shown in **Figure 8**. Research hotspots on macrophage polarization in AS evolved from “alternative activation” in 2009 to “oxidative stress” and “coronary heart disease” in 2021.

### Co-Cited Reference and Reference Burst

Co-citation is the frequency with which two documents are cited together (15). **Table 6** displays the top 10 co-cited references in which co-citation occurred at least 37 times. “Distribution of macrophage polarization markers in human atherosclerosis” (16), authored by Stoger (a member of de Winther’s team) and published in *Atherosclerosis* was the most co-cited reference in macrophage polarization in AS (26), followed by a review article entitled “Macrophage Phenotype and Function in Different Stages of Atherosclerosis” (27) (18). In total, 7 of the top 10 most highly co-cited papers were review articles, and 3 were experimental studies, including “Human Atherosclerotic Plaque Alternative Macrophages Display Low Cholesterol Handling but High Phagocytosis Because of Distinct Activities of the PPARγ and LXRα Pathways” (28) (25) and “Distribution of macrophage polarization markers in human atherosclerosis” (16),

**Table 6:** Top 10 co-cited references for macrophage polarization research in AS.

Rank	Author	Title	Journal	Centrality	Co-Citation
1	Stoger JL (2012) (16)	Distribution of macrophage polarization markers in human atherosclerosis	Atherosclerosis	0.04	67
2	Tabas I (2016) (17)	Macrophage Phenotype and Function in Different Stages of Atherosclerosis	Circulation Research	0.05	64
3	Moore KJ (2013) (18)	Macrophages in atherosclerosis: a dynamic balance	Nature Reviews Immunology	0.02	63
4	Chinetti-Gbaguidi G (2015) (19)	Macrophage subsets in atherosclerosis	Nature Reviews Cardiology	0.11	61
5	Khallou-Laschet J (2010) (20)	Macrophage Plasticity in Experimental Atherosclerosis	Plos One	0.04	57
6	Murray PJ (2014) (21)	Macrophage Activation and Polarization: Nomenclature and Experimental Guidelines	Immunity	0.06	51
7	Moore KJ (2011) (22)	Macrophages in the Pathogenesis of Atherosclerosis	Cell	0	48
8	Leitinger N (2013) (23)	Phenotypic Polarization of Macrophages in Atherosclerosis	Arteriosclerosis Thrombosis and Vascular Biology	0.02	38
9	Mantovani A (2009) (24)	Macrophage Diversity and Polarization in Atherosclerosis	Arteriosclerosis Thrombosis and Vascular Biology	0.02	38
10	Chinetti-Gbaguidi G (2011) (25)	Human Atherosclerotic Plaque Alternative Macrophages Display Low Cholesterol Handling but High Phagocytosis Because of Distinct Activities of the PPARγ and LXRα Pathways	Circulation Research	0.08	37

**Figure 9:**
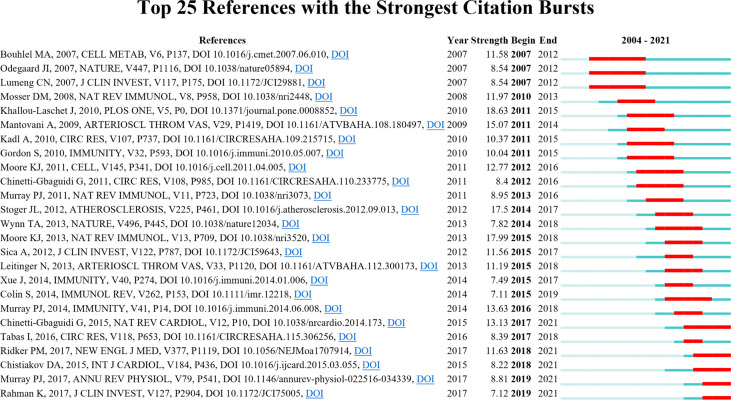
Top 25 references with the citation bursts (sorted by the beginning year of the burst). The blue bars mean the reference had been published; the red bars mean citation burstness.

As shown in **Figure 9**, CiteSpace detected 25 references with the most substantial citation bursts. The earliest reference with citation bursts was from 2007 to 2012, entitled “PPARγ Activation Primes Human Monocytes into Alternative M2 Macrophages with Anti-inflammatory Properties” by Bouhlel et al. (29) and published in *Cell Metabolism* and “Macrophage Plasticity in Experimental Atherosclerosis” by Khallou–Laschet (20) et al. and published in *Plos One*, which had the strongest burstness (strength = 18.63).

## Discussion

### General Information of Main Findings

In 2004, only three articles were published regarding studies of macrophage polarization in AS (30–32). As of 2010, the number of research studies remained low. The year 2011 was a turning point for this subject, as more researchers began focusing on macrophage polarization in AS and published related articles with a rapid upward trend. From 2015 to 2021, annual outputs entered a stable growth phase. AS with macrophage polarization research showed a wave-like uplift trend that is likely to increase in the future.

In the authors and co-cited authors analysis, de Winther, a researcher from the University of Amsterdam and the University of Munich, made the most contributions with 17 published studies, followed by Staels from INSERM and the University of Lille Nord de France with 10 articles. It is important to note that de Winther’s team focused on explaining the mechanisms of macrophages in AS. In recent years, “Epigenetic,” (33), “genome-wide association study” (34), and “single-cell analysis” (35) were the primary directions of their research. After constructing bone marrow of myeloid Kdm6b-deficient mice model, de Winther’s team found that insufficient epigenetic enzyme Kdm6b accelerated AS progression, although the content was similar in all groups. Their latest study found that ATP citrate lyase is an activator of plague vulnerability, and targeting therapy of macrophage metabolism suggests pathways for AS treatment (36). Mantovani was the most co-cited author, and his team had a significant influence in this field (21).

The United States, China, Germany, Netherlands, and Japan were the top five productive countries/regions. The US was the first country to explore macrophage polarization in AS (2004), followed by Italy (2005), Japan (2007), France (2009), and Canada (2009). It is noteworthy that China began studies later (2013); however, China ranked second in output. Among the top 10 countries/regions, the USA, Germany, Italy, and England shared “bridge” roles in research. Although China was the second highest publication country, the relatively low centrality (0.08) suggests that Chinese researchers should broaden their international cooperation to deepen their impact. Amsterdam University (Netherlands), Maastricht University (Netherlands), and Harvard University (USA) published the highest number of papers. Harvard University and Maastricht University have made outstanding contributions in this field.

As shown in **Tables 3**, **4**, *Arteriosclerosis Thrombosis and Vascular Biology* published the most papers and had the most significant number of co-cited journals over 2000, implying a significant role in the research area. *Atherosclerosis*, *Frontiers in Immunology*, and *Plos One* were also productive journals related to macrophage polarization in AS. Papers published in high IF journals, such as *Circulation Research*, *Journal of Immunology*, *Circulation*, and *Nature*, had more co-citations and might provide a theoretical basis for future research.

### Knowledge Base of Macrophage Polarization in AS

Co-citation analysis is a measure that reveals the intrinsic patterns of a research literature category over some time, and studies with high co-citations are often considered to be the research basis in a field (37). Literature co-citation is a feature function of CiteSpace and was one of the first to be used and theoretically discussed when CiteSpace was developed (38). The top 10 co-cited references are described below.

Maastricht University researcher Stoger collaborated with eight other researchers and published the highest co-cited research in *Atherosclerosis* in 2012 (16). They applied transcriptomic and immunohistochemistry methods to elucidate the dynamics of macrophage phenotype in successive stages of AS. M1 and M2 macrophages accumulate as plaques progress. M1 cells predominate over M2 polarized cells in the rupture-prone shoulder region of the plaque, whereas the fibrous cap of the lesion does not differ significantly between the subsets. CD163 is associated with hemorrhagic plaques. However, foam cells showed an ambiguous cell convergence that could also detect M1 and M2 markers.

The second most co-cited article was a review article systematically summarizing the subsets and functions of macrophages in AS stages by Tabas in 2016 (17). This review discussed macrophage phenotypic transformation from the perspective of body microenvironment, intracellular lipid metabolism, pro-inflammatory, and pro-lysis mediator balance and suggested a bidirectional relationship between macrophage metabolic state and phenotype.

Moore et al. (18) found that macrophages in plaques were in a dynamic equilibrium stage. Their team discussed recent discoveries in identifying inflammation responses relating to lipid metabolism and potential therapeutic targets of macrophages.

In 2015, Chinetti-Gbaguidi et al. published a review article in *Nature Reviews Cardiology* (19). They listed the macrophage subtypes contained in plaques, subsets activation conditions, and macrophage phenotype markers in humans and mice and explained the role of macrophage responses to hemorrhage, cytokines, growth factors, and roles in plaque vulnerability.

The fifth most co-cited paper was published by Khallou-Laschet et al. in *Plos One* 2010 (20). An ApoE knockout mice model was constructed to evaluate macrophage subtypes transformation in AS. M1 and M2 were labeled by arginase (Arg) II and Arg I. The authors found that M2 cell infiltration was predominant in the initial stage of AS, activating smooth muscle cell proliferation, whereas M1 macrophages were predominant in aged-knockout mice. Polarized macrophages still retained their plasticity.

Murray et al. (21) proposed a common macrophage nomenclature framework from three principles for the controversial description of macrophage polarization and made preliminary recommendations for reproducible experiment criteria and the minimum reporting standards. This review attempted to provide a standard for future studies of macrophage polarization in AS. The seventh most co-cited article was also published by Moore et al. (22) in *Cell* (2011). It discussed the specific pathogenesis of macrophages in AS-related diseases such as stroke, myocardial infarction, and sudden cardiac death and summarized the principles of treating these diseases. Leitinger et al. (23) summarized and analyzed relevant studies before 2013 through phenotypic and functional characteristics of macrophages, aiming to provide the basis for future drug intervention strategies.

Mantovani et al. (24) elucidated the mechanism in AS from the perspective of macrophage homeostasis, on the basis of a summary of studies before 2009. Chinetti-Gbaguidi et al. (25) identified a unique subtype of macrophages, CD68+ mannose receptor (MR)+ M2, in human atherosclerotic plaques, showing low sensitivity to transform to foam cells but with high phagocytic activity.

Among the top 10 co-cited articles, the review and experiment categories reflect the field’s knowledge base, summarizing the basic and clinical research findings over time and providing guidance and evidence for future research.

### Identification of Research Hotspots and Emerging Topics

In bibliometrics, keyword co-occurrence analysis reflects an academic subject’s hotspots and research trends; a cluster of keywords analysis shows the knowledge structure, and the timeline view visualizes the keyword hotspot evolution (39). “Inflammation,” “monocyte,” “gene expression,” “NF kappa b,” “disease,” and “foam cell” shared the primary research directions in macrophage polarization in AS.

From the visualization of keywords-cluster analysis, “m1,” “macrophage polarization,” “adipose tissue,” “NF kappa b,” “scavenger receptor,” “activated macrophage,” “mice,” “metabolic reprogramming,” “MAPK,” and “vascular biology” constructed the knowledge structure. Several factors influence macrophage polarization, including lipid metabolism and glycolysis (28, 40). Lipid metabolism is a significant contributor to atherosclerotic plaque progression. Circulating monocytes differentiate into foam cells by taking up lipid and ox-LDL and forming lipid encapsulated bodies (foam cells) (41). Foam cells lead to inflammatory mediator secretion in plaques, accelerating AS progression. Scavenger receptors and CD36 rely on Lyn to internalize ox-LDL to prompt foam macrophage formation and M1 macrophage polarization (41, 42). You et al. (43) found that sorting nexin 10, a regulator of lipid metabolism in macrophages, induced macrophage reprogramming, and promoted AS development through the Lyn-Akt/TFEB pathway.

The keywords foam cell (32), smooth muscle cell, LDL (32), and oxidative stress appeared in 2004, indicating the initial research emphasis. From 2005 to 2010, the keywords inflammation (44), disease (31), nitric oxide, alternative activation, endothelial cell (22, 45), adipose tissue (46), PPAR-gamma, NF kappa b, and immune response enriched the original research topics. In the last 10 years, as scholars explored macrophage polarization in AS, studies related to coronary heart disease (47), metabolism, apoptosis (48), myocardial infarction (49), Toll-like receptor, HDL, pathogenesis, cholesterol efflux (50), therapeutic target, and nanoparticle became the new hotspots. As seen from the timeline analysis, studies expanded from learning the initial mechanisms to exploring therapeutic targets for relevant diseases. Inflammatory responses, foam cell formation, endothelial cell dysfunction, and lipid metabolism have been the core of research contents in this field. Cholesterol efflux, shear stress, fatty acid oxidation, and nanoparticle (51) studies are new frontiers of research.

Citation Burst analysis detects emerging dynamic concepts and potential research questions in a field and is suitable for examining emerging trends and sudden changes in disciplinary development, reflecting active or cutting-edge research nodes (52). In keywords citation burst analysis, keywords of alternative activation, dendritic cell, T cell, smooth muscle cell, and adipose tissue were suddenly increased from 2009 to 2014. From 2015 to 2017, IFN-gamma, recruitment, lipopolysaccharide, tumor necrosis factor-alpha, fatty acid oxidation, rheumatoid arthritis, hypercholesterolemia, *in vivo*, and tumor-associated macrophage were critical research directions. From 2018 to 2021, the keywords of the research hotspots evolved to adipose tissue macrophage, lipid metabolism, collagen, risk, *in vitro*, oxidative stress, coronary heart disease, and prevention.

Reference citation burst analysis also characterizes the emerging topics of a subject. The most substantial citation burst article came from a landmark study by Khallou-Laschet et al. (20) in 2011 (18.63, 2011–2015), which experimentally explained the distribution and polarization of M1 and M2 macrophages in plaques. Moore et al. (18) published the second citation burst review in *Nat Rev Immunol* in 2013 (17.99), whose burst began in 2015 and ended in 2018. In this review, the authors elucidated the roles of macrophages in plaque evolution and inflammatory pathways relating to lipid efflux, guiding future research.

The references with citation bursts in 2007 focused on obesity-related macrophage polarization. PPARγ was positively correlated with anti-inflammatory M2 macrophage expression in human atherosclerotic plaques (29). Odegaard et al. (53) used PPARγ-deficient mice to show that PPARγ was necessary to transform alternative-activated macrophages. Disruption of PPARγ in bone marrow cells impairs alternative macrophage activation, promoting these animals to develop diet-induced obesity, insulin resistance, and glucose intolerance. In an obesity mice model, M2 macrophages polarized to M1 may contribute to insulin resistance (54).

Among the top 25 references with the strongest citation bursts, five are still in the burst phase today. These articles represent the latest emerging topics of macrophage polarization in AS, which suggests future potential research directions. A review article published by Chinetti-Gbaguidi et al. (19) in 2015 had the highest burst strength (13.13), which began from 2017 (2017–2021), providing an overview of macrophage subsets in plaque progression and pathology. A randomized, double-blind trial conducted by Ridker et al. (55) of canakinumab (a monoclonal antibody against IL-1β) treated patients with previous myocardial infarction and high-sensitivity c-reactive protein level of 2 mg or higher per liter. Anti-inflammatory therapy targeting IL-1β at 150 mg every 3 months resulted in a significant reduction in the recurrence of cardiovascular events but not in reducing lipid levels. This study was published in 2017 in *The New England Journal of Medicine* with the citation burst strength of 11.63; the burst began in 2018 and lasted until this writing. Chistiakov et al. (56) published a review in the *International Journal of Cardiology* in 2015 (strength = 8.22, 2018–2021). This review discussed the distribution of macrophage phenotypes in atherosclerotic plaques and the role of lipids and transcription factors on macrophage phenotype modifications.

In 2017, Murray et al. (57) published another influential review article (strength = 8.81, 2019–2021) in the *Annual Review of Physiology*. This article explained the instability and plasticity of macrophage polarization, which integrates research results up to 2017. It summarized the molecular-level mechanisms of macrophage polarization, differences in macrophage polarization between humans and mice, macrophage survival, expression of signature genes, and development of drugs to regulate polarization, including CSF-1 inhibitors.

Rahman et al. (58) found that, in WT, CD68− GFP reporter, Apoe^−/−^, Ccr5^−/−^, Ccr2^−/−^, Cx3cr1^−/−^, or Stat6^−/−^ mice with aortic arch transplantation, Ly6C^hi^ monocytes were essential for plaque regression and inflammation elimination. The authors also suggested that relevant clinical treatment strategies to enhance M2 polarization in atherosclerotic plaques might serve as methods for plaque regression.

We conclude that the hotspots of macrophage polarization in AS initially focused on its impact on obesity. Studies explored more critical targets in this area, including Nrf2 (59), Kruppel-like factor 4 (60–63), GLP-1/GLP-1R (64), TLR4 (27, 65), and micro RNA (66, 67), related to inflammation process and plaque progression. Cardiovascular disease studies, including the role of macrophage polarization in lipid metabolism, inflammatory immune response in plaques, and cerebrovascular disease, are two diseases involved in this field. Therapeutic method exploration related to these two diseases will emerge as research goals in the future. Clinical trials with anti-inflammatories for atherosclerotic disease treatment have been conducted; however, the evidence from these studies remains insufficient.

### Limitations

Data were downloaded from the WoSCC database; therefore, studies not collected in WoSCC were missed. However, WoSCC is the most commonly used database in scientometric analysis and includes most information in related articles. At the beginning of the study, we simultaneously used other strategies such as Medline to search the references and found that the number of related studies was relatively low. Therefore, we finally choose WoSCC to conduct this research. The uneven quality of the data collected in the studies might impair the credibility of knowledge mapping. Bibliometric applications might lead to bias, as reported in other bibliometric studies (26). Nevertheless, the visualization-based literature analysis sets the stage for researchers to understand the hotspots and potential trends in macrophage polarization in AS.

## Conclusion

We gathered general information and created a knowledge base of the internal structure, hotspot evolution, and emerging topics in macrophage polarization in AS studies from 2001 to 2021. The studies of macrophage polarization in AS have outstanding research value and application prospects. CiteSpace and VOSviewer bibliometric analysis showed a significant trend in this area. The leading countries concerned with this topic are the United States and China; however, cooperation and communication among countries and institutions need to be strengthened. While focusing on basic research, researchers should pay attention to translating results to clinical work. Inflammation has been a core element throughout the research in this field. The research in this field is focused on the mechanism research, targeted therapy, and biomarkers in cardiovascular and cerebrovascular diseases; these will be critical subjects in future studies.

## Data Availability Statement

The original contributions presented in the study are included in the article/supplementary material. Further inquiries can be directed to the corresponding author.

## Author Contributions

LS conceived the study. JZ, DM, and YF searched and downloaded the data. WT, RL, and ZZ re-examined the data. LS, JJ, and JZ analyzed the data. LS drafted the manuscript. JJ, DM, and HX reviewed the manuscript. All authors contributed to the article and approved the submitted version.

## Funding

China’s National Natural Science Foundation supported the work (no. 82074215).

## Conflict of Interest

The authors declare that the research was conducted in the absence of any commercial or financial relationships that could be construed as a potential conflict of interest.

## Publisher’s Note

All claims expressed in this article are solely those of the authors and do not necessarily represent those of their affiliated organizations, or those of the publisher, the editors and the reviewers. Any product that may be evaluated in this article, or claim that may be made by its manufacturer, is not guaranteed or endorsed by the publisher.
